# Transmission patterns of smallpox: systematic review of natural outbreaks in Europe and North America since World War II

**DOI:** 10.1186/1471-2458-6-126

**Published:** 2006-05-05

**Authors:** Vibha Bhatnagar, Michael A Stoto, Sally C Morton, Rob Boer, Samuel A Bozzette

**Affiliations:** 1 Health Services Research and Development, Veterans Affairs San Diego Healthcare System, San Diego, California, USA; 2 Department of Family and Preventive Medicine, University of California San Diego, San Diego, California, USA; 3 The RAND Center for Domestic and International Health Security, Arlington, Virginia, USA; 4 The RAND Center for Domestic and International Health Security, Santa Monica, California, USA; 5 Department of Public Health, Erasmus MC, University Medical Center Rotterdam, The Netherlands

## Abstract

**Background:**

Because smallpox (variola major) may be used as a biological weapon, we reviewed outbreaks in post-World War II Europe and North America in order to understand smallpox transmission patterns.

**Methods:**

A systematic review was used to identify papers from the National Library of Medicine, Embase, Biosis, Cochrane Library, Defense Technical Information Center, WorldCat, and reference lists of included publications. Two authors reviewed selected papers for smallpox outbreaks.

**Results:**

51 relevant outbreaks were identified from 1,389 publications. The median for the effective first generation reproduction rate (initial R) was 2 (range 0–38). The majority outbreaks were small (less than 5 cases) and contained within one generation. Outbreaks with few hospitalized patients had low initial R values (median of 1) and were prolonged if not initially recognized (median of 3 generations); outbreaks with mostly hospitalized patients had higher initial R values (median 12) and were shorter (median of 3 generations). Index cases with an atypical presentation of smallpox were less likely to have been diagnosed with smallpox; outbreaks in which the index case was not correctly diagnosed were larger (median of 27.5 cases) and longer (median of 3 generations) compared to outbreaks in which the index case was correctly diagnosed (median of 3 cases and 1 generation).

**Conclusion:**

Patterns of spread during Smallpox outbreaks varied with circumstances, but early detection and implementation of control measures is a most important influence on the magnitude of outbreaks. The majority of outbreaks studied in Europe and North America were controlled within a few generations if detected early.

## Background

The anthrax attacks that followed the events of September 11th, 2001 focused attention on the threat of terrorism with biological agents including variola major, the causative agent of smallpox [1]. There is concern that an attack with smallpox could result in many deaths because of the susceptibility of the U.S. population [2-8]. A number of issues, such as the amount of virus released and the number of people exposed, need to be considered in order to estimate the impact of a bioterrorist attack with smallpox. However, one key factor in determining the size of an outbreak resulting from such an attack is the transmissibility of smallpox, which can be difficult to estimate. One measure of transmissibility is the effective reproductive rate (R), which is equal to the expected number of infections produced by an infectious host in a real world setting [5,9-11].

R can be determined in a straightforward manner for individual epidemics and is related to the circumstances, such as population vaccination status, under which the individual epidemics occur. When considering intentional attacks, the greatest interest lies in initial spread, much of which often occurs before an outbreak is recognized. For this reason, we focused on the transmission from index cases to the first generation of secondary cases, which we termed the initial R.

The data for this study was obtained from a systematic literature review for reports of smallpox outbreaks. We limited our review to reports of smallpox outbreaks occurring in Europe and North America after 1945 because these societies were much more similar to the modern United States in terms of their demographic, social, and physical structure, than were the pre-World War II western and the developing societies from which most smallpox outbreak reports have emanated. Moreover, population immunity was also relatively more similar to a contemporary US population than might be expected *a priori*. While early post-war European and North American populations were generally vaccinated in childhood, re-vaccination rates were generally low and few adults had more than remote vaccination [12,13]. Immunity naturally acquired through infection was also similarly low in both populations because outbreaks were rare. Although terrorist use of smallpox in the 21^st ^century may have substantially different epidemiological characteristics (see discussion below), the outbreaks analyzed in this paper are the closest actual population experience that may help partially guide modern smallpox control efforts.

## Methods

### Data collection

**Figure 1:**
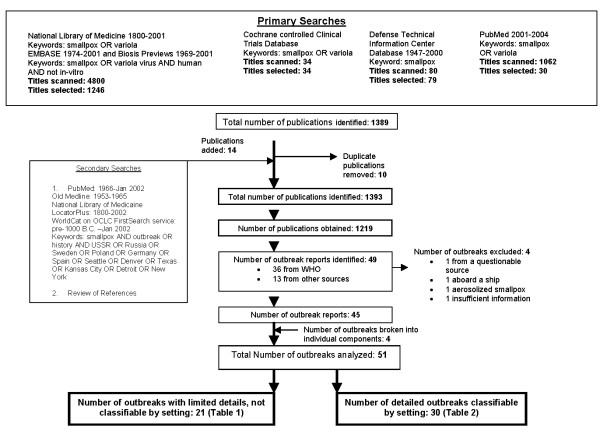
Literature Search. 1389 publications were selected and 45 post World War ll outbreaks were identified for analysis.

The following databases were searched for literature on smallpox through 2001: National Library of Medicine 1800 to 2001 (keywords: smallpox or variola), Embase 1974 to 2001, Biosis 1969–2001 (keywords: smallpox or variola virus and human and not in-vitro), Cochrane Controlled Clinical Trials Library (keywords: smallpox or variola) and Defense Technical Information Center 1947 to 2000 (keywords: smallpox). The search was later updated by a PubMed search from 2001 to 2004 (keywords: smallpox or variola). Duplicate titles were removed. Secondary searches included more specific searches for outbreaks in specific countries and sites, and a review of reference lists from publications identified in other searches (Figure 1). There was no language restriction.

Two authors (SB and VB) independently scanned titles for relevance, and publications chosen by either reviewer were reviewed further. One of the authors (VB) scanned the selected articles to identify post-1945 smallpox outbreaks from Europe and North America. Two abstractors (RB or research associate and VB) independently extracted data from the identified outbreaks. Discrepancies between reviewers were resolved by consensus. Non-English publications were abstracted by one author (RB) or by a research assistant under an author's (VB) direction.

To be included in the analysis, the following had to be available: the date of occurrence, and the number of index cases, total cases, cases in each generation, and generations. The following outbreak details were also extracted if available: the number of cases acquired within a hospital, a household, or at a distance (cases not acquired by household or hospital contact); the number of missed cases (cases not diagnosed as smallpox at the time of illness) and deaths; the generation at which the outbreak was identified, and the clinical presentation of the index case. Index cases were classified as having had typical smallpox, atypical smallpox (a milder illness as seen in people with prior smallpox vaccination and/or a rash not characteristic of smallpox), or hemorrhagic smallpox (a usually fatal illness characterized by internal hemorrhage and/or petechiae) [14]. The following data regarding the efficacy of control measures were also extracted if available: the use of ring vaccination (the vaccination of people who were in close contact with a patient with smallpox), case isolation (the separation of suspected cases of smallpox from the general public), quarantine (the restriction of movement in or out of a region that has been exposed to smallpox), mass vaccination (the vaccination of an entire region), and/or prophylactic vaccinia immune globulin administration [15-17].

### Analysis

The number of outbreaks over the period studied was small (total of 45 outbreaks) and the circumstances varied widely. Therefore, a descriptive and qualitative analysis was emphasized. For this analysis, the initial R was measured by dividing the total number of first generation cases by the number of index (zero^th ^generation) cases. In addition, several outbreaks had sufficient details to be classified by setting. Because hospitals were often the centre of outbreaks, setting was defined according to the proportion of secondary cases (non-index cases) acquired within a hospital [12,13,18-20]. After examination of the distribution of case locations across outbreaks, hospital outbreaks were defined those in which 70% or more of secondary cases were hospital acquired, mixed outbreaks as those in which 30% to 70% of secondary cases were hospital-acquired, and community outbreaks as those in which 30% or less of secondary cases were hospital-acquired.

## Results

### Literature search

Based on the results of the primary searches, 5,976 titles were scanned; 1,389 of these were selected. Despite initial de-duplication, 10 of these were duplicates (Figure 1). Secondary searches identified 14 additional publications. Of the resulting 1,393 publications, 1219 (87%) were obtained; 92 journal citations (7% of the selected publications) and 41 government reports (3% of the selected publications) were unobtainable.

Forty-nine post-1945 European and North American outbreaks were identified. Thirty-six of these were included in a single World Health Organization (WHO) publication [21] and prior work by T.M. Mack [12]. Several outbreaks were documented in multiple publications (Tables 1 and 2). Four outbreaks were excluded. The source of the first outbreak could not be confirmed as the outbreak may have resulted from the importation of smallpox on a rug (Union of Soviet Socialist Republics or U.S.S.R, 1959). The second and third outbreaks occurred under unique circumstances; one occurred aboard a ship (Poland, 1962) and the other may have been the result of testing aerosolized smallpox (U.S.S.R, 1972) [21-23]. A fourth outbreak was excluded because the number of cases in each generation was not documented; therefore, the initial reproductive rate (R) could not be derived from the data (United States or U.S., 1946) [24]. The remaining 45 original outbreaks occurred over a thirty-year period in different countries and under different circumstances. The majority of reported outbreaks occurred in three countries: 18 (40%) in the United Kingdom (U.K.), 8 (18%) in the Federal Republic of Germany (F.R.G.), and 3 (7%) in the U.S.S.R. (Tables 1 and 2). Most resulted from involved index cases imported from endemic countries, but two outbreaks were the result of laboratory exposure [25,26].

Although published or listed as single outbreaks, four outbreaks included multiple epidemiologically distinct components, such as occurred when a case travelled to a different region resulting in a secondary outbreak in the new area (see reference column and footnotes for Table 2). The components of these four outbreaks were treated as 10 separate outbreaks so that an effective total of 51 outbreaks were analyzed [21,27-29]. For all 51 outbreaks, available information included the total number of generations and cases, the number of cases in each generation, the duration of the outbreak, and the number of deaths (Tables 1 and 2). Thirty of the outbreaks could also be categorized by setting and had detailed information on the generation that the outbreak was identified, control measures, clinical presentation of the index case, and the number of missed cases (Table 2).

### Characteristics of the 51 outbreaks

**Figure 2:**
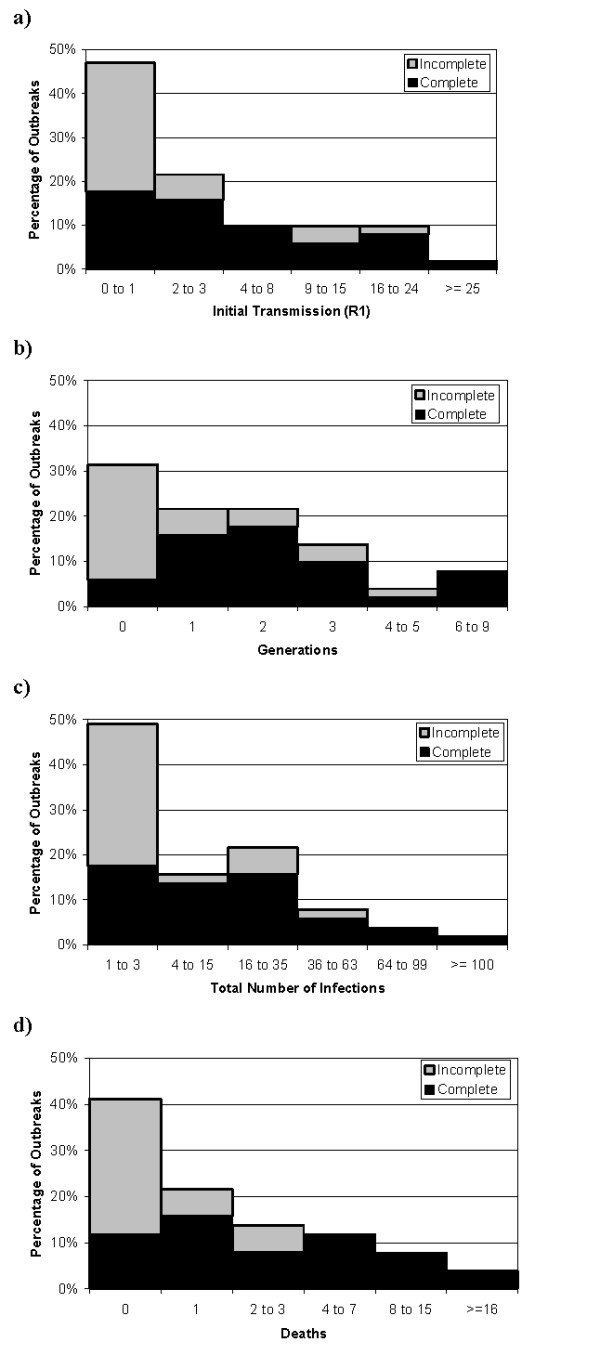
**Characteristics of European and North American Smallpox Outbreaks**. Characteristics of outbreaks reported in detail compared to incompletely reported outbreaks: initial reproductive rate (Figure 2a), outbreak duration (Figure 2b), outbreak size (Figure 2c), and number of deaths (Figure 2d). Dark bars: characteristics of 30 outbreaks reported in detail. Light bars: characteristics of 21 incompletely reported outbreaks.

The median initial reproduction rate (R) across all 51 outbreaks was 2 with a range of 0 to 38 (Table 3, Figure 2a). About half had an initial R of 1 or less, and over two-thirds had an initial R of 3 or less. The median duration, as measured by number of generations, was 1 with a range of 0 to 9 (Table 3, Figure 2b). About a third did not extend beyond the index generation, and nearly three quarters lasted for 3 or fewer generations. The median outbreak size, as measured by the total number of cases, was 4 with a range of 1 to 134 cases (Table 3, Figure 2c). About half involved 3 or fewer cases, and two-thirds involved 15 or fewer cases. The median number of deaths was 1 with a range of 0 to 26 (Table 3, Figure 2d). About two-fifths involved no deaths, and three quarters involved 3 or fewer deaths.

Outbreaks with greater initial R-values tended to be larger and longer. For example, in outbreaks with an initial R of 5 or less versus more than 5, median values for the total number of cases and generations were 2 versus 23 and 1 versus 2.5 respectively.

### Characteristics of the 30 outbreaks reported in detail

There was no temporal trend in the amount of detail reported, but the thirty outbreaks reported in detail typically had a larger R (median 2 vs.0), lasted more generations (median 2 vs. 0), and had more total cases (median 13 vs. 1) and deaths (median 2 vs. 0) compared to less-detailed outbreaks (Table 3, Figures 2a–d). In the detailed outbreaks, most of the index cases had atypical (mild) or hemorrhagic disease. Because these clinical presentations of smallpox are less common, many of these index cases were missed or not diagnosed with smallpox at the time of illness. The majority of index cases were infected because of travel in smallpox endemic countries, and most countries required a certificate of vaccination for travel. Two of the index cases were infected as a result of laboratory exposure [25,26]. All of these 30 outbreaks were managed by ring vaccination and case isolation. Although a few outbreaks used additional control measures, we are unable to evaluate the efficacy of these other strategies. These included mass vaccination (used in New York City, U.S., 1947 and Kosovo, Yugoslavia, 1972), quarantine (used in Kosovo, Yugoslavia, 1947) and administration of Vaccinia Immune Globulin (used in Meschede, Germany, 1969) [20,21,30,31].

### Initial reproduction rate

**Figure 3:**
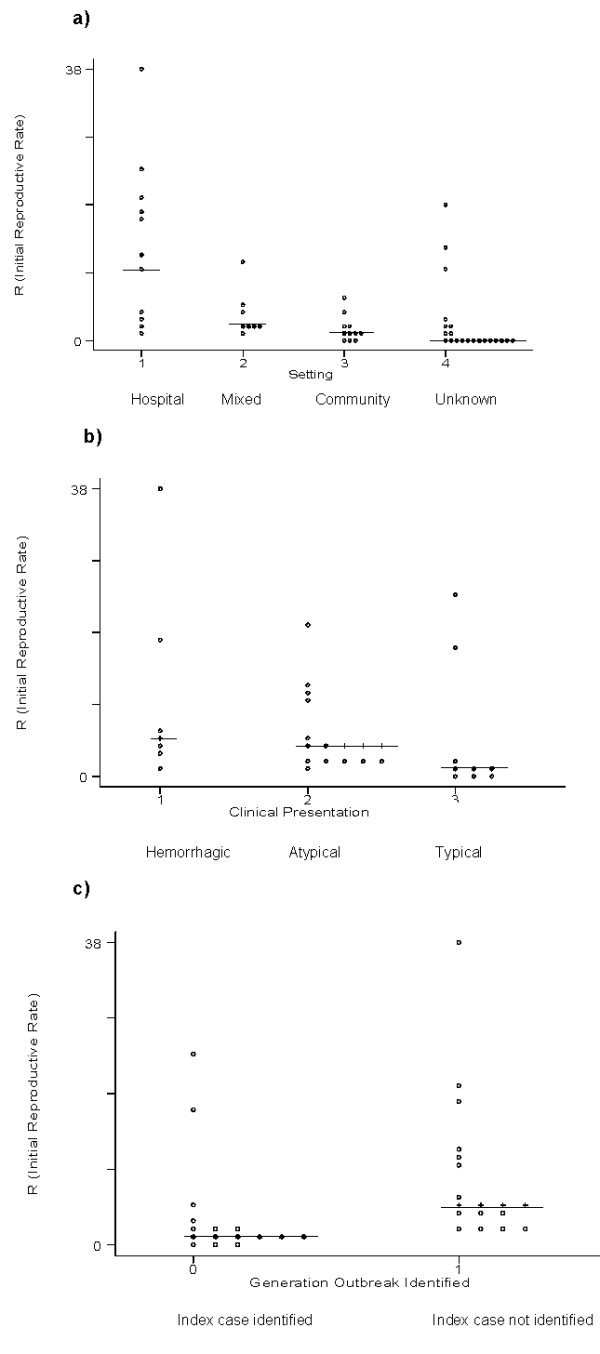
**Initial Reproductive Rate (Median ---)**. Initial effective reproduction rate (initial R) in 30 post-World War II Western European and North American smallpox outbreaks by setting (Figure 3a), clinical presentation of the index case (Figure 3b), and identification of the index case with smallpox (Figure 3c).

The median initial R across the 30 detailed outbreaks was 2 with a range of 0 to 38 (Table 3, Figure 2a). About a third had an initial R of 1 or less, and about two thirds had an initial R of 3 or less. Outbreaks that remained in the community had a lower initial R (median 1) than those that were hospital based from the first generation (median 12) (Table 3, Figure 3a). Mixed outbreaks, which generally started in the community and later spread through hospital contacts, had an intermediate initial R (median 2) (Table 3, Figure 3a). The initial R was smaller when the index case(s) had a typical versus atypical or hemorrhagic presentation (medians 1 vs. 4 or 5, respectively), and when the index case was identified as smallpox versus unrecognized (median 1 vs. 5) (Table 3, Figures 3b and 3c). The effect was most pronounced in hospital outbreaks – median initial R's for identified versus unidentified index cases in hospital outbreaks were 3 versus 15, but the same values for community and mixed outbreaks were 1 versus 4, and 2 versus 2, respectively.

### Other characteristics

The median duration for 30 outbreaks was 2 generations with a range is 0 to 9 generations (Table 3, Figure 2b). About a third did not extend beyond the first generation, and another third lasted for only through the second generation. Outbreaks that remained in the community lasted a median of 1 generation, while those that were hospital-based lasted a median of 2 generations and those that were mixed lasted a median of 3 generations (Table 3). Outbreaks tended to be shorter when the index case was typical compared to atypical or hemorrhagic (median number of generations 1 vs. 3 vs. 2, respectively) and when the index case was identified (median number of generations 1 vs. 3) (Table 3). The duration of hospital outbreaks was not affected by the identification of the index cases, but the duration of mixed and community outbreaks lasted up to 6 and 9 generations, respectively, when index cases were unidentified.

The median outbreak size, as measured by the total number of cases, was 13 with a range of 1 to 134 cases (Table 3, Figure 2c). There were approximately equal proportions of small (total number of cases less than 5) and large (total over 20) outbreaks (Table 2). Outbreaks that remained in the community involved a median of 2 cases, while those that were hospital based involved a median of 20 cases and those that were mixed had a median of 22 cases (Table 3). Outbreaks tended to be smaller when the index case was typical versus atypical or hemorrhagic (median number of cases 2 vs. 27 vs. 16.5) and when the index case was identified (median number of cases 3 vs. 27.5) (Table 3). The outbreak size was similarly small across all 3 settings when the index case was identified and similarly large across the settings when it was not.

The median number of deaths was 2 with a range of 0 to 26 (Table 3, Figure 2d). About one-fifth involved no deaths, and four fifths involved 4 or fewer deaths (Table 2). Community, hospital based, and mixed outbreaks had medians of 1, 4, and 3 deaths, respectively (Table 3); however, case fatality rates were 0.24, 0.20 and 0.17. The number of deaths was smaller if the index case was identified (median equals 1 vs. 6, Table 3). The case fatality rate was highest if the index case presented with hemorrhagic-type smallpox.

There was a strong association between typical presentation and early identification of index cases: index cases were identified as smallpox in 9 of 9 outbreaks when the presentation was typical, 3 of 13 when it was atypical, and 2 of 6 when it was hemorrhagic. This relationship aids in the understanding of outbreak severity. For example, 93% of outbreaks had greater than 10 cases when the index case was not identified. Moreover, in all of these larger outbreaks, the index cases were atypical or hemorrhagic. When the index case was identified, most outbreaks had less than 10 cases regardless of whether the index case was typical, atypical, or hemorrhagic.

## Discussion

Descriptions of smallpox transmission are important to biosecurity planning, especially since some planning efforts have posited outbreaks resulting in numbers of cases – hundreds, thousands, or more – that are very large relative to the clinical experience shortly before eradication of the natural disease [32]. In a systematic review designed to provide an understanding of smallpox transmission patterns, we identified and analyzed what were effectively 51 smallpox outbreaks from post-1945 Europe and North America. In these outbreaks, we found a median of 4 total cases and 1 death.

The characteristics of the outbreaks varied greatly: one outbreak with an initial R of 11 involved 134 people and caused 26 deaths over only 3 generations of spread; another outbreak with initial R of only 2 involved 28 cases but lasted 9 generations. However, most outbreaks were small and contained within a few generations: 31 percent involved only one case, 41 percent caused no deaths, and 31 percent had an initial R of 0 (i.e., no first generation cases).

Examination of outbreaks within categories of setting, identification of the index case, and clinical characteristics provided additional insights. By reviewing original publications, we were able to categorize and analyze many outbreaks by setting. For example, were able to review the original publications for eight outbreaks documented by the WHO [25,26,28,33-37] as well as, identify thirteen additional outbreaks [20,27,29-31,38-41]. Several of these outbreaks also had epidemiologically distinct components, resulting in 51 effective outbreaks for analysis.

Many (six out of eleven) of the hospital outbreaks were only found in the above-mentioned additional outbreaks [20,27,29,30,38,39]. Hospital outbreaks had higher initial reproductive rates than either community or mixed outbreaks, and had more cases and deaths than did community types. Moreover, the interaction of identification and setting in the studied outbreaks reveals a pattern similar to that seen in frequently unrecognized infectious diseases, such as tuberculosis and, more recently, SARS [42]. When index cases were identified, control is established early and both the median initial R and the number of generations were similarly low in all 3 settings. When the index cases were unidentified, a large increase in the median initial R was seen only in the hospital setting and a large increase in the number of generations was seen only in community and mixed settings. This is consistent with the observation that, within the close quarters of an institution, the transmission of smallpox is relatively effective before identification and containment measures are quite effective after identification. This impression is reinforced by examination of the two shipboard outbreaks that were excluded from the analysis. In these outbreaks, the initial R's were 6 and 25, respectively, but recognition was followed by control within two generations [22].

In community outbreaks, transmission is intrinsically less effective. The consequence of missed initial identification in the community was not an elevated initial R, but an increase in the length of the outbreaks. The longer outbreaks may be due to a decrease in the effectiveness of containment measures after initial spread in the community, possibly because of the greater difficulty of tracking and managing cases in that setting. Nonetheless, all of the 30 outbreaks reported in detail were brought under control, generally by case isolation and ring vaccination. Other measures were rarely used (mass vaccination was used in the New York City, 1947 outbreak and mass vaccination and quarantine were used in the Kosovo, 1972 outbreak [21,30]) and there is limited information on the efficacy of these additional measures. It should also be noted that newer treatment modalities, such as Cidofovir, have been shown to be protective in animal studies against vaccinia and other orthopox viruses (monkeypox and cowpox) after exposure [43,44].

Outbreaks beginning with an atypical or hemorrhagic versus a typical presentation of the index case had more cases, a higher initial transmission rate, and were somewhat longer. This is related to the ease of initial diagnosis: only non-typical cases were mis-identified, and outbreaks in which the index case was mis-identified were generally larger, deadlier, longer, and had a median initial R of 5 compared to 1 if the case was identified. We speculate that the large difference in initial R is because, when the index case is identified, this parameter is effectively the initial reproductive rate with early institution of control measures. However, it is clear that the types of outbreaks seen in developed countries in the second half of the 20th century were well and quickly controlled.

Despite searching several databases, using broad searches, scanning nearly 6,000 titles, and reviewing papers in several languages, our requirement that included epidemics involve post-World War II western populations limited us to identifying only 45 separate outbreaks, some of which were divided for analysis. The majority of the outbreaks were from only three countries, suggesting that outbreaks from certain countries were more likely to have been documented. The 30 outbreaks reported in detail were larger than the 21 that were briefly reported; it is probable that smaller outbreaks were not routinely published in detail because outbreaks were still relatively common and smallpox was still endemic in many parts of the world. This apparent publication bias, however, suggests that there may have been many unpublished small outbreaks and that this analysis may overestimate the transmission potential of smallpox because it is based only on published reports.

The recent literature contains several estimates for the reproductive rate of smallpox. It is difficult to directly compare our findings and estimates for initial R, the initial effective reproduction rate for smallpox, to these other studies. Two of the studies do not use contemporary western outbreaks. One estimates the basic reproductive rate, (R_0, _a theoretical parameter defined as the expected number of new infected hosts that an infectious host will produce in a large randomly mixed population of susceptible individuals [9]), rather than effective reproduction rate. Specifically, Gani and Leach used epidemic modelling of data from 19th Century European smallpox outbreaks to estimate a R_0 _of 3.5 to 6.0 [19]. Eichner and Dietz obtained an R of 6.9 from data on a 1967 outbreak in Nigeria [5]. Meltzer et al examined post-1961 outbreaks from countries around the world in estimating an R of 3 [8].

Our estimate of the overall initial R is quite comparable to Meltzer et al., but use of any single point estimate for R_0 _or initial R in policy analysis ignores a main lesson of this review. This work clearly shows that the pattern, speed, and duration of an outbreak's spread vary widely according to the specific circumstances surrounding an outbreak. Different smallpox release scenarios should be expected to yield different results in part because the parameters and modified natural history of the outbreak itself will vary with the scenarios. For example, our previous work modelled the outcome of terrorist attacks under different scenarios [4]. These scenarios included community based outbreaks resulting from infected cases riding a busy transit system and the intentional release of aerosolized smallpox in airport terminals, and a mixed outbreak resulting from the release of aerosolized smallpox in a large office building. Although adjustments had to be made for a poorly vaccinated population and healthcare workforce, the understanding of smallpox transmission patterns in hospital, mixed and community settings, provided important insights into how smallpox may spread under different attack scenarios.

In a contemporary US population, one might expect transmission from introduced smallpox to be higher than we found to be typical. Population density is higher; mobility is greater, and, though more similar that one might think, immunity in the contemporary U.S. population is likely somewhat lower than in the populations studied. The healthcare workforce is also poorly vaccinated and inexperienced with smallpox. This will decrease the human resources available for care and containment, and increase the likelihood that initial cases will be missed. Counterbalancing this is the fact that few index cases will be vaccinated recently so the likelihood of more easily recognized typical cases is much higher. As the outbreak scenarios of greatest concern involve intentional release, outbreaks could be larger and more obvious than those reviewed, or could involve strains selected or engineered to be more virulent or contagious.

Finally, the outbreak from Kosovo deserves notice because this region is not as developed or as wealthy as most of the other countries studied. This outbreak was the largest identified for this review (N = 176 for both combined hospital and mixed components). Moreover, the hospital and mixed component of this outbreak had the second highest (R = 38) and highest (R = 11) initial reproductive rate, and the hospital component lasted 9 generations (median number of generations for a hospital outbreak = 2). This suggests that a smallpox outbreak in a less developed country with limited resources for healthcare, disease surveillance, and case isolation could be potentially more devastating than a bioterrorist attack in a Western/industrialized country [44].

## Conclusion

It is clear that most post-World War II outbreaks in Western countries were small and had low transmission rates. This was almost uniformly the case when the index case presented with typical smallpox and was recognized early. However, exceptions were common and heterogeneous, particularly when (usually atypical or hemorrhagic) index cases were not initially recognized. Initially unrecognized community outbreaks lasted several generations despite low initial R values. This suggests that control measures were less effective in this setting, perhaps because of the difficulty tracing and vaccinating all cases and contacts. In contrast, unrecognized hospital outbreaks were controlled quickly despite high initial R values, suggesting that control measures worked very well in a closed, contained setting. Early detection, particularly of patients who do not present with typical smallpox, coupled with early implementation of control measures decreases both the duration and size of outbreaks in all settings.

## Competing interests

The author(s) declare that they have no competing interests.

## Authors' contributions

VB managed and conducted the systematic review and data analysis. She was primary author of this manuscript. MAS was a senior policy advisor, and helped with project management and data analysis. SCM was a senior biostatistical advisor and consultant for the literature review. RB helped conduct the literature review and data analysis. SAB was the senior investigator who designed, obtained funding and coordinated this project. All authors were involved in drafting this paper and have approved the final manuscript.

## Funding Sources

RAND Corporation, Department of Veterans Affairs

## Pre-publication history

The pre-publication history for this paper can be accessed here:



## Figures and Tables

**Table 1:** Outbreaks reported with fewer details, not classifiable by setting

	**YEAR**	**COUNTRY, CITY**	**REFERENCE**	**INITIAL R**	**TOTAL CASES**	**NUMBER OF GENERATIONS**	**NUMBER OF DEATHS**
**1**	1958	Fed. Rep. Germany, Heidelberg	[21]	10	20	3	2
**2**	1959	United Kingdom, Liverpool	[21]	0	1	0	0
**3**	1959	German Dem. Rep., Berlin	[21]	0	1	0	0
**4**	1959	USSR, Moscow	[21]	19	46	3	3
**5**	1960	United Kingdom, London	[21]	0	1	0	0
**6**	1961	Spain, Madrid	[21]	13	17	2	3
**7**	1961	Fed. Rep. Germany, Ansbach	[21]	2	4	2	1
**8**	1961	USSR, Moscow	[21]	0	1	0	0
**9**	1961	Belgium, Brussles	[21]	0	1	0	1
**10**	1961	USSR, Kirovabad	[21]	0	1	0	0
**11**	1961	Fed. Rep. Germany, Lammersdorf	[21]	3	33	4	1
**12**	1962	United Kingdom, London	[21]	2	3	1	0
**13**	1963	Poland, Gdansk	[21]	0	1	0	0
**14**	1963	Switzerland, Zurich	[21]	0	1	0	0
**15**	1967	Fed. Rep. Germany, Regensburg	[21]	1	2	1	0
**16**	1967	Czechoslovakia, Prague	[21]	0	1	0	0
**17**	1967	Fed. Rep. Germany, Hanover	[21]	0	1	0	0
**18**	1967	United Kingdom, London	[21]	1	2	1	0
**19**	1968	United Kingdom, London	[21]	0	1	0	0
**20**	1968	Belgium, Namur	[21]	0	1	0	0
**21**	1973	United Kingdom, London	[21]	0	1	0	0

**Table 2:** Outbreaks reported with more details, classifiable by setting

	**YEAR**	**COUNTRY, CITY**	**REFERENCE(S)**	**PROPORTION OF CASES ACQUIRED WITHIN A HOSPITAL **	**INITIAL R**	**TOTAL CASES**	**NUMBER OF DEATHS**	**NUMBER OF GENERA-TIONS**	**GENERATION OUTBREAK IDENTIFIED**	**NUMBER OF CASES NOT INITIALLY DIAGNOSED (MISSED CASES)**	CLINICAL PRESENTATION OF INDEX CASE*
HOSPITAL OUTBREAKS											
**1**	1947	United States, New York	[30]	0.91	4	12	2	2	1	1	Hemorrhagic
**2**	1950	United Kingdom, Glasgow	[29]	1.00	20	21	3	1	1	1	Atypical
**3**	1952	France, Marseille(1)	[27]	0.97	24	40	4	3	0	0	Not clear from text^a^
**4**	1954	France, Vannes	[27, 38]	0.88	12	74^b^	16	3	1	2	Atypical
**5**	1961	United Kingdom, Bromwich	[21, 28]	1.00	1	2	0	1	0	0	Typical
**6**	1961	United Kingdom, Bradford	[21, 28, 34]	1.00	10	14	6	2	1	1	Atypical
**7**	1962	United Kingdom, Cardiff (2)	[21, 28]	1.00	18	21	13	2	1	1	Hemorrhagic
**8**	1963	Poland, Lodz(3)	[39]	1.00	3	4	1	1	0	0	Hemorrhagic
**9**	1969	Meschede	[20, 31]	1.00	17	20	4	2	0	0	Typical
**10**	1972	Yugoslavia, Kosovo (4)	[21]	0.86	38	43	9	2	1	38	Hemorrhagic
**11**	1973	United Kingdom, London	[21, 25]	1.00	2	4	2	2	0	0	Typical
MIXED OUTBREAKS											
**12**	1947	United Kingdom, Barnsley	[29]	0.38	5	17	0	3	0	1	Atypical
**13**	1949	United States, Hidalgo County	[40]	0.33	2	8	1	2	1	5	Atypical
**14**	1950	United Kingdom, Brighton	[29]	0.46	2	29	10	4	1	3	Atypical
**15**	1952	France, Marseille(1)	[27]	0.50	1	3	1	2	0	0	Not clear from text^a^
**16**	1961	Dusseldorf	[21, 35]	0.50	2	5^c^	2	2	0	0	Typical
**17**	1963	Poland, Wroclaw (3)	[21, 33]	0.48	2	90	6	6	2	4	Atypical
**18**	1963	Sweden, Stockholm	[21, 36]	0.44^d^	4	27	4	6	2	5	Atypical
**19**	1972	Yugoslavia, Kosovo (4)	[21]	0.36	11	134	26	3	2	12	Atypical
COMMUNITY OUTBREAKS											
**20**	1947	United Kingdom, Bilston	[29]	0.04	2	28	1	9	1	3	Atypical
**21**	1953	United Kingdom, Industrial Pennines	[29]	0.23	4	39	11	6	1	8	Atypical
**22**	1961	United Kingdom, London	[21] [28]	0.00	2	3	1	1	0	0	Atypical
**23**	1962	United Kingdom, Birmingham	[21] [28]	0.00	0	1	0	0	0	0	Typical
**24**	1962	United Kingdom, Cardiff (2)	[21] [28]	0.00	1	2	1	1	0	1	Typical
**25**	1962	Canada, Toronto	[21]	0.00	0	1	0	0	0	0	Typical
**26**	1962	United Kingdom, Cardiff (2)	[21] [28]	0.17	6	25	6	3	1	1	Hemorrhagic
**27**	1963	Poland, Opole (3)	[41]	0.00	1	4	0	1	0	0	Atypical
**28**	1965	Fed. Rep. Germany, Kulmbach	[21] [37]	0.00	0	1^e^	0	0	0	0	Typical
**29**	1970	Denmark, Skodsborg	[21]	0.00	1	2	1	1	0	0	Hemorrhagic
**30**	1978	United Kingdom, Birmingham	[26]	0.00	1	2	1	1	0	0	Typical

* Atypical: index cases with a milder illness (usually seen with a previous vaccination for smallpox) or a rash not characteristic of smallpox. Detailed outbreaks (from Table 2) analyzed by components:

(1) Outbreak from Marseille, France (1952) broken into two components: Entries 3 and 15 [27].

(2)Outbreak from Cardiff, U.K.(1963)broken into three components: Entries7, 24 and 26 [21, 28].

(3) Outbreak from Poland (1963) broken into three components: Entries 8, 17 and 27 [41].

(4)Outbreak from Kosovo, Yugoslavia (1972) broken into two components: Entries 10 and 19 [21].

Footnotes for Table 2:

a Nature of the index cases was not clear from the text (limited information) [27].

b Total number of cases listed as 74, consistent with text [38] and second reference [27].

c Total number of cases listed as 5 [28, 34] ; 6 is listed in World Health Organization Table 23.4 [21].

d Proportion of hospital acquired cases listed as 0.44 because three nurses infected as a result of household exposure and were setting excluded as hospital contacts [21, 36].

e Total number of cases listed as 1 [37]; 2 is listed in World Health Organization Table 23.4 [21].

**Table 3:** Summary of parameters for 51 post-World War II Western European and North American smallpox outbreaks.

	Number of Outbreaks Identified	Median for Initial R	Median for Number of Generations	Median for Number of Total Cases	Median for Number of Deaths
Overall	51	2	1	4	1
Reporting Status					
Incompletely reported	21	0	0	1	0
Reported in detail	30	2	2	13	2
Outbreak Setting					
Community	11	1	1	2	1
Mixed	8	2	3	22	3
Hospital	11	12	2	20	4
Clinical Presentation of Index Case					
Hemorrhagic	6	5	2	16.5	4
Atypical*	13	4	3	27	4
Typical	9	1	1	2	1
Index Case Identified as Smallpox					
No	14	5	3	27.5	6
Yes	16	1	1	3	1

* Atypical: index cases with a milder illness (usually seen with a previous vaccination for smallpox) or a rash not characteristic of smallpox.
